# Prevalence of Cardiac Amyloidosis in Patients Referred for Transcatheter Aortic Valve Replacement

**DOI:** 10.1016/j.jacc.2017.11.037

**Published:** 2018-01-30

**Authors:** Paul R. Scully, Thomas A. Treibel, Marianna Fontana, Guy Lloyd, Michael Mullen, Francesca Pugliese, Neil Hartman, Philip N. Hawkins, Leon J. Menezes, James C. Moon

Aortic stenosis (AS) is the most common valve disease in the developed world. Symptomatic severe AS has poor outcomes unless treated. Transcatheter aortic valve replacement (TAVR) numbers are growing fast. Amyloidosis is caused by the deposition of abnormally folded protein resulting in progressive organ dysfunction. Wild-type transthyretin amyloid (wtATTR) affects the heart, causing a restrictive cardiomyopathy—deposits can be found in up to 25% of individuals >85 years of age at autopsy 1, 2. Until recently, diagnosis required endomyocardial biopsy. However, bone scintigraphy (^99m^Tc-3,3-diphosphono-1,2-propanodicarboxylic acid [DPD], ^99m^Tc-pyrophosphate [PYP], or ^99m^Tc-hydroxymethylene diphosphonate [HMDP]) is remarkably sensitive at detecting cardiac transthyretin amyloid (ATTR) (1). This has led to the development of formalized guidelines, enabling noninvasive diagnosis (1). Castaño et al. (3) recently reported occult cardiac ATTR in 16% of patients post-TAVR. We investigated the coexistence of cardiac ATTR in patients with severe AS before undergoing TAVR.

**Figure 1:**
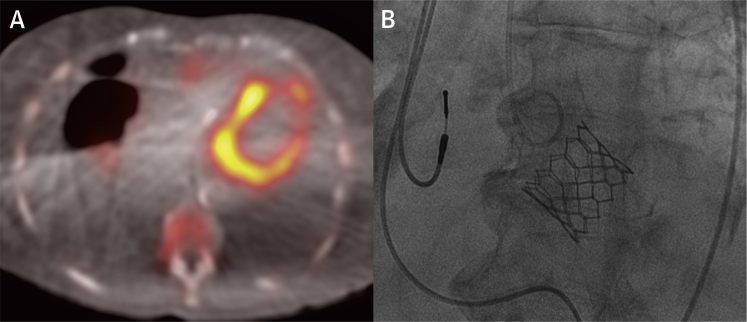
Cardiac Amyloid in Aortic Stenosis An 88-year-old woman with severe aortic stenosis. **(A)** DPD scintigraphy fused single-photon emission tomography/computed tomography illustrating cardiac tracer retention suggestive of cardiac amyloid, with large bilateral pleural effusions. **(B)** Fluoroscopy demonstrating the 26-mm Edwards Sapien 3 valve (Edwards Lifesciences, Irvine, California) in situ during the transcatheter aortic valve replacement. Note the pre-existing dual-chamber pacemaker leads.

We conducted a pre-specified futility interim analysis of the ATTRact-AS study (NCT03029026), which seeks the prevalence and impact of occult cardiac amyloid in the elderly (aged 75 years and over) with severe AS. The bone tracer was DPD. Scans were graded using planar and single-photon emission tomography/computed tomography images (Figure 1) by 2 experienced observers and reviewed by the National Amyloidosis Centre (NAC), United Kingdom. If positive (Perugini grades 1 to 3), primary light chain (AL) amyloidosis was excluded by blood and urine monoclonal immunoglobulin testing. As per protocol, clinicians were informed pre-TAVR only if there was a possibility of AL amyloidosis. After TAVR, all DPD-positive patients were referred to the NAC.

We recruited 101 patients (mean age 86 ± 5 years, 43% male) who underwent DPD scintigraphy between October 2016 and August 2017, representing a quarter of patients on the Barts Heart Centre TAVR pathway. Echocardiographic baseline findings were: *aortic valve:* peak velocity 4.19 ± 0.68 m/s, peak gradient 72 ± 23 mm Hg, mean gradient 43 ± 15 mm Hg, mean area by continuity equation 0.71 ± 0.22 cm^2^, mean area indexed to body surface area (BSA) 0.40 ± 011 cm^2^/m^2^; *left ventricle:* mean ejection fraction 54 ± 11%, mean stroke volume indexed to BSA 38 ± 10 ml/m^2^, mean septal thickness 13 ± 2 mm (range 7 to 21 mm).

Cardiac ATTR was diagnosed in 14 patients (13.9%, 95% confidence interval: 8% to 22%). Perugini grade was 1 (n = 4) and 2 (n = 10). In this subgroup, 50% were male, with a mean age of 88 ± 6 years. The mean aortic valve gradient and stroke volume indexed to BSA were slightly lower in the DPD-positive cohort (37 ± 12 mm Hg vs. 44 ± 15 mm Hg, and 32 ± 7 ml/m^2^ vs. 38 ± 11 ml/m^2^); however, this did not reach statistical significance (p = 0.11 for both).

Three DPD-positive patients had a plasma cell dyscrasia, but after review at the NAC, AL amyloidosis was felt unlikely in all cases. All DPD-positive patients genotyped so far were wild type (n = 5). There was 1 periprocedural permanent pacemaker, 1 implantable cardiac defibrillator, and 1 spinal cord infarction in the DPD-positive cohort. There were also 2 deaths pre-TAVR in the DPD-positive cohort (14%).

These findings support the work of Castaño et al. (3) that approximately 1 in 7 patients currently undergoing TAVR have occult cardiac amyloidosis—a higher prevalence than surgical aortic valve replacement cohorts (4). We note that 2 DPD-positive patients died before TAVR, raising the possibility that these patients represent a higher-risk subgroup even pre-procedure. Furthermore, wtATTR typically affects males more, but here the male and female prevalence of amyloid were similar (16% prevalence in men, 12% in women), as found in the heart failure with preserved ejection fraction population (5). Finally, the amyloid burden was skewed: rather than a pyramidal distribution with DPD grade 1>2>3, DPD grade 2 was dominant. This may support proposals of an interaction between AS and amyloid.

The confirmation of such a high prevalence of occult amyloid in TAVR patients has implications. Cardiac amyloid profoundly alters the myocardium and is likely to affect clinical presentation and outcomes (procedural approaches, benefits, mortality). The importance of ongoing studies is high.
